# The cost and utilisation patterns of a pilot sign language interpreter service for primary health care services in South Africa

**DOI:** 10.1371/journal.pone.0189983

**Published:** 2017-12-22

**Authors:** Tryphine Zulu, Marion Heap, Edina Sinanovic

**Affiliations:** 1 Health Economics Unit, School of Public Health and Family Medicine, Health Sciences Faculty, University of Cape Town, Cape Town, South Africa; 2 Health and Human Rights Programme, School of Public Health and Family Medicine, Health Sciences Faculty, University of Cape Town, Cape Town, South Africa; Universita degli Studi di Perugia, ITALY

## Abstract

**Background:**

The World Health Organisation estimates disabling hearing loss to be around 5.3%, while a study of hearing impairment and auditory pathology in Limpopo, South Africa found a prevalence of nearly 9%. Although Sign Language Interpreters (SLIs) improve the communication challenges in health care, they are unaffordable for many signing Deaf people and people with disabling hearing loss. On the other hand, there are no legal provisions in place to ensure the provision of SLIs in the health sector in most countries including South Africa. To advocate for funding of such initiatives, reliable cost estimates are essential and such data is scarce. To bridge this gap, this study estimated the costs of providing such a service within a South African District health service based on estimates obtained from a pilot-project that initiated the first South African Sign Language Interpreter (SASLI) service in health-care.

**Methods:**

The ingredients method was used to calculate the unit cost per SASLI-assisted visit from a provider perspective. The unit costs per SASLI-assisted visit were then used in estimating the costs of scaling up this service to the District Health Services. The average annual SASLI utilisation rate per person was calculated on Stata v.12 using the projects’ registry from 2008–2013. Sensitivity analyses were carried out to determine the effect of changing the discount rate and personnel costs.

**Results:**

Average Sign Language Interpreter services’ utilisation rates increased from 1.66 to 3.58 per person per year, with a median of 2 visits, from 2008–2013. The cost per visit was US$189.38 in 2013 whilst the estimated costs of scaling up this service ranged from US$14.2million to US$76.5million in the Cape Metropole District. These cost estimates represented 2.3%-12.2% of the budget for the Western Cape District Health Services for 2013.

**Conclusions:**

In the presence of Sign Language Interpreters, Deaf Sign language users utilise health care service to a similar extent as the hearing population. However, this service requires significant capital investment by government to enable access to healthcare for the Deaf.

## Introduction

The World Health Organisation estimates disabling hearing loss to be around 5.3% [1], while a study of hearing impairment and auditory pathology in the Limpopo, South Africa found a prevalence of nearly 9% [2]. Communication between a health provider and the patient is a pre-requisite for any meaningful intervention to occur in the process of seeking health care. Amongst the signing Deaf people, however, communication difficulties represent the extreme in the continuum of communication challenges experienced in health care interactions [3]. The use of English as the main language of communication in health care further exacerbates this challenge in South Africa, as most Deaf people have limited access to health literacy and low levels of English comprehension [4,5]. Only 20% of deaf adults in the USA demonstrate fluency in written English and the average English reading level among Deaf high school learners is at or below the 4th Grade [6]. Glaser and Lorenzo report similar findings for South Africa[7]. Literacy affects academic achievement. In South Africa, it was as late as 1968 that the first Deaf people–from Worcester–matriculated. The level of matriculation and Grade 12 obtained, unfortunately rarely offered University entrance. At the end of the 1990s it was estimated that there were only 15 Deaf people in South Africa with university degrees [8].

Studies from western countries including the UK, have revealed disparities in health outcomes between the hearing and the Deaf population with the Deaf exhibiting poorer health[9]. In Africa, these disparities have been studied in the field of HIV/AIDS where prevalence rates have been shown to be higher amongst the Deaf and hard of hearing than the population average [10]. This is largely due to a lack of knowledge on prevention and poor access to care due to inability to communicate with staff, marginalisation and the social construct that sees deaf people as asexual [10]. Prioritising communication needs of the Deaf ensures that signing Deaf patients benefit from health systems’ initiatives designed to promote both preventative and curative health services [6].

Communication methods commonly utilised in the health services include ad hoc interpreters such as family or friends and the use of written instructions[5,11,12]. Barriers to effective communication using a family member include a lack of objectivity and impartiality, unfamiliarity with medical jargon, and a breach of the patient’s privacy where the patient might not be willing to share their medical problem, [13], particularly important in HIV/AIDS [14]. In the broader lower income categories, the use of a relative to provide interpretation may delay consultation as the patient relies on the availability of third parties to access care. Health expenditure may rise substantially as the indirect costs of seeking health care on the household increase because of the need for a “double” consultation per family. Using written communication is not effective given the low literacy levels amongst Deaf patients [15].

In view of the above, SLIs have been identified as an important human resource in health care in order to afford better health care access and improve the health care experiences of the Deaf. However, provision of SLIs is sporadic in most countries and is often seen as the responsibility of the Deaf patient more than of the health systems [12,16]. This inability of health systems to accommodate the Deaf and hard of hearing through provision of SLIs undermines the role of health systems as forces of social cohesion and weakens the role of patients as “co-producers of health” [17]. Consequently, it has been argued that provision of SLIs should be viewed not only from a human rights perspective but also as a risk mitigation strategy given the deleterious effects of miscommunication between health care providers and patients such as adverse reactions and over dosage [13]. In South Africa this has been identified as warranting attention by the South African National AIDS Council, which deems the prioritisation of funding to enable communication in multiple languages, including Sign Language, to be important in curbing the spread of HIV/AIDS through improved communication and access to treatment. [18]. However, in order to realise the goals of SLI provision, policy makers require cost estimates hence the present study.

Accredited South African Sign Language Interpreters’ fees range from US$25.94 to US$36.31 per hour exclusive of VAT, plus an additional US$0.23 per kilometre for transport [19]. This makes it out of reach of most Deaf patients who are often unemployed and depend on state-sponsored grants [4]. Further it has been shown that non-statutory funding of Deaf initiatives is unsustainable and also results in inequity [20], hence the need for a tax-funded SASLI program.

Information on the use of professional sign-language interpreters in the South African health services context is limited and there is also unavailability of published data on the socio-economic status of the deaf and their utilisation rates of health care services in South Africa. It is against this background that the study seeks to describe this group’s health care utilisation patterns, quantify the costs and to ascertain the budgetary impact and affordability of running the South African SLI project, with the aim of advocating for the up-scaling of the service to district health services to alleviate these communication barriers.

This study draws on an on-going pilot project housed at the UCT’s School of Public Health and Family Medicine. It analyses the costs and utilisation patterns of the SASLI service in health care provided free-to-patient for Deaf clients accessing health services within the Cape Metro as well as training medical SLIs.

## Materials and methods

### i) Costing

This was a retrospective costing analysis done using both financial and economic costs at the project level from a provider perspective to calculate unit costs per interpreter-assisted visit for 2013. A visit in the public sector facilities is more of an ‘encounter’ that involves queuing at different points in the facility for up to 6–7 hours. These include security checks, reception (file retrieval), waiting room queue for routine tests (such as urine, bloods, blood pressure), nurses' consultation, doctors’ consultation, filling a prescription at the pharmacy and next appointment booking. An ingredients approach was utilised, taking into account all inputs that go into making a single SASLI–assisted visit possible. The unit costs calculated from the project level were then used to estimate the costs of scaling up the service to the Cape Metropole District Health Services. Capital and recurrent costs data was obtained from the pilot project. Interviews were conducted with a field expert on training of interpreters in order to obtain the true cost of training a Sign Language interpreter (S1 Table). All costs are reflected in 2013 US$.

Capital costs considered were office space, furniture, equipment and training costs. The replacement cost of the furniture and equipment was used in the calculation, with a lifespan of 10 years and 5 years respectively [21]. Recurrent costs included personnel, operating costs such as rent, water and electricity, transport and office consumables. Overhead costs were calculated using the allocation factor based on the proportion of the building occupied by the SASLI Project. Personnel costs were split according to the proportion of time the interpreters spend on each of the tasks as laid out in the job description.

### ii) Socioeconomic and demographic data

A secondary data analysis was done on the socio-economic profile of 136 Deaf respondents who had previously used the project or were likely clients for the project. The data extracted included average monthly income, employment status, gender and the highest level of education obtained. For all SASLI-assisted visits, information on the type and location of health care facility visited was also extracted.

### iii) Data analysis

The costing data was analysed in Microsoft Excel 2010 whilst the utilisation data was analysed using Stata v.12. The total costs at the project level were added up and divided by the total number of interpreter-assisted visits for 2013. The costs in South African Rands were converted to US$ using the average exchange rate prevailing in 2013 from www.oanda.com, (US$1 = ZAR 9.6388).

### iv) Estimating the costs of scaling up

In order to calculate the costs of providing the SASLI service per annum at the district level the following variables were utilised (Table 1):

1. Average utilisation rate of the SASLI service per person per year (calculated at the pilot project level based on utilisation data from 2008 to 2013 (S2 Table)).
2. Estimated population in need i.e. the number of SASL users (based on the DeafSA statistics assuming all signing Deaf people will need an interpreter).
3. The cost per SASLI assisted visit (calculated at the pilot project level).

**Table 1:** Parameters used for the calculation of the costs of scaling up.

Variable	Quantity	Sources of data
Total South African Population (2011 census)	51 800 000	Statistics South Africa
Cape Metro District population (2011 census)	3 740 026	Statistics South Africa
High estimate of SASL users in South Africa	1 500 000	DeafSA
High estimate of SASL users in the Cape Metro District	108 302	Proportion based on DeafSA StatisticsSA and census data
Low estimates of SASL users in South Africa	600 000	DeafSA
Low estimates of SASL users in the Cape Metro District	43 321	Proportion based on DeafSA StatisticsSA and census data
Western Cape Provincial Health Budget (2013/2014)	US $1.656 billion	www.westerncape.gov.za
District Health Services Allocation (2013/2014)	US $626.3 million	www.westerncape.gov.za
Average utilisation rate per person per year (lowest)	1.66	Calculated from study
Median utilisation rate per person per year	2	Calculated from study
Average utilisation rate per year per person (highest)	3.58	Calculated from study
Exchange rate	US $1 = ZAR9.6388	www.oanda.com/currency/

### v) Sensitivity analysis

Sensitivity analysis was carried out on the personnel and discount rate variables. The calculations were done using permanently employed full-time interpreters as opposed to the ad hoc contracting. The discount rate was also changed to 6% to ascertain the impact on the costs per visit.

### Ethics

The University Of Cape Town’s Human Research Ethics Committee (HREC) approved this study with reference HREC 618/2013. This study draws on the secondary data of four related but independent stand-alone studies. No primary data was collected from human subjects specifically for this study. The University of Cape Town’s Human Research Ethics Committee approved the four studies with individual reference numbers i.e. HREC 428/2006, HREC 043/2011, HREC 044/2011, and HREC 193/2013 respectively. In all the four studies, the study participants gave written consent with a signed consent form. The information pamphlet was available in South African Sign Language in the form of a DVD and in a written form in three local spoken languages of the Western Cape–English, Afrikaans and isiXhosa. The consent form was also made available in these three local spoken languages. This consent procedure was approved by the HREC.

## Results

### i) Utilisation of SASLI

**Fig 1:**
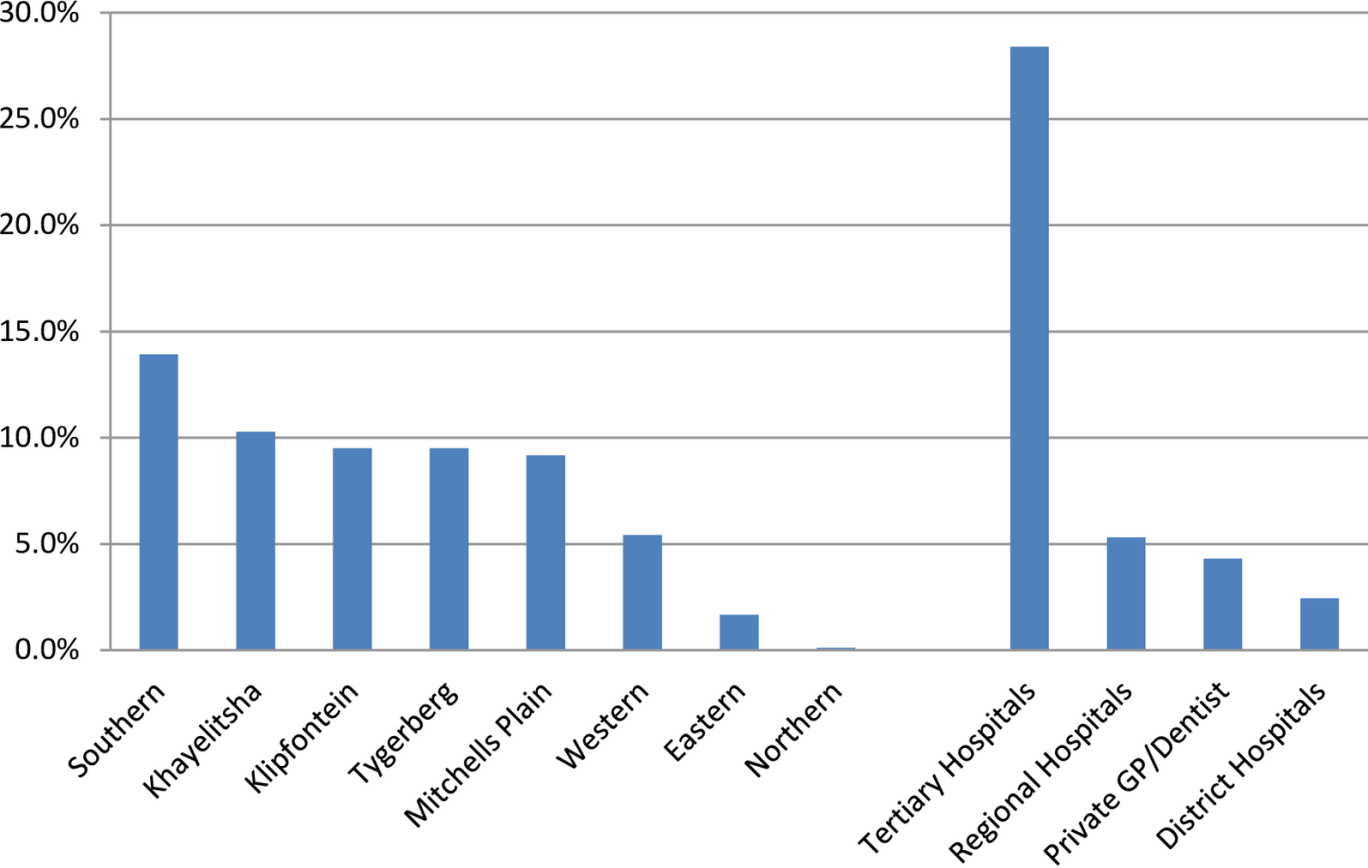
Sign Language Interpreter-assisted health care visits to sub district primary healthcare facilities and hospitals (2008–2013).

The average number of requests per Deaf person ranged from 2.6 ± 2.2 to 4.02 ± 4.44 requests per year while actual mean utilisation per person ranged from 1.66± 1.32 to 3.58± 3.61 per year between 2008 and 2013. Of the total number of requests between 2008 and 2013, 159 (15.9%) did not get an interpreter. However this proportion declined steadily from 31.8% in 2008 to 10% by 2013, largely due to training of more interpreters and hiring of more ad hoc interpreters. The distribution of facilities for which an interpreter was utilised is shown by geographic location and type of facility in **Fig 1.**

### ii) Demographic and socio-economic characteristics of the Deaf respondents

The average age of the respondents was 40.5 ± 11.3 years with an age range of 20 to 70 years (N = 136). Sixty-two percent (n = 84) of the respondents were female. The vast majority (74%, n = 61) of the employed earned less than US$416.40 per month and only one person earned more than US$1041. A summary of the socio-economic and demographic variables is shown in Table 2.

**Table 2:** The demographic and socio-economic profile of the Deaf respondents.

	Frequency (N = 136)	Proportion
**Language**		
Afrikaans	23	16.9%
English	99	72.8%
Xhosa	14	10.3%
**Marital Status**		
Divorced	12	8.8%
Married	49	36.0%
Single living alone	15	11.0%
Single living with family	24	17.6%
Single living with partner	24	17.6%
Single living with people—not family	4	2.9%
Widow/Widower	8	5.9%
**Education Level**		
Below Grade 7 or equivalent	51	37.5%
Between Grade 7 and Grade 12 (or equivalent)	74	54.4%
Passed matric	6	4.4%
Have post-matric qualification	3	2.2%
Don’t know	2	1.5%
		
**Employment status**		
Employed	81	59.6%
Unemployed	55	40.4%
		
**Monthly Income**		
Not disclosed	3	2.2%
No Income	22	16.2%
Pension/social grant	31	22.8%
< US$415	60	44.1%
US$415 –US$1037	19	14.0%
> US$1037	1	0.7%
Average age	40,5 ± 11,3	
Age range	20–70 years	

### iii) Cost per visit

There were a total of 326 interpreter-assisted visits in 2013 with an average cost of $189.38 per visit, of which personnel costs were 64%; capital costs 24% and operating costs 12% (Table 3). The training cost, as part of capital costs, is the equivalent of the course fees, which also includes trainers’ fee, venue, and printing of manuals and materials. Extrapolating to the district level using the estimates of the population in need from DeafSA and the utilisation rate calculated from the pilot project (Table 1) gave cost estimates of between US$13.6 million and US$73.4 million in 2013 (Table 4**)**.

**Table 3:** Breakdown of provider costs in 2013 US$.

Cost type	Unit cost/visit	Proportion of total costs
**Recurrent Cost**		
Personnel	$121.12	64.0%
Operating costs	$22.64	12.0%
**Capital Costs**		
Training	$29.56	15.6%
Building	$10.50	5.5%
Equipment	$4.73	2.5%
Furniture	$0.83	0.4%
**Total cost per visit**	**$189.38**	

**Table 4:** Cost of SASLI services as a proportion of the District and Provincial health budgets (in 2013 US $).

Annual Utilisation rate	Low estimates of population in need			High estimates of population in need		
	Estimated cost of SASLI in Health (million)	% of *DHS Budget	% of **Prov. Health Budget	Estimated cost of SASLI in Health (million)	% of *DHS budget	% of **Prov. Health Budget
**1.66**	**13.62**	2.2%	0.8%	**34.04**	5.4%	2.1%
**2**	**16.41**	2.6%	1.0%	**41.02**	6.5%	2.5%
**3.58**	**29.37**	4.7%	1.8%	**73.43**	11.7%	4.5%

*DHS- District Health Services

** Provincial Health Services

### iv) Sensitivity analysis

Using permanently employed fulltime senior interpreters and junior interpreters, as opposed to the trainee SASLIs from the project, increased the cost per SASLI assisted visit by 74% at the project level from US$189.38 to US$329.52. Using a discount rate of 6% increases the cost per interpreter-assisted visit at the project level marginally to US$194.56 from US$189.38 per visit.

## Discussion

The increasing utilisation of SASLI services indicates that the pilot project has been able to mitigate the health care access needs of the Deaf in a significant way. Average utilisation rates of interpreters as a proxy for health care utilisation from this study are closer to the targeted national average of 3.5 and higher than the actual Western Cape Provincial Department of Health utilisation rate of 2.6 visits per capita. This validates the fact that health care needs and utilisation of healthcare services of the Deaf are no different from the hearing population given adequate access to SLI. The average utilisation rate of 3.58 per person per year is also similar to that seen in Sweden (3.6), which has a comparatively well-developed SLI service [16]. Admittedly, this increased utilisation of health care services by the Deaf in the presence of an interpreter will lead to an increase in health care costs in the Cape Metropole of between 2.2% and 11.7% of district health services budget depending on the estimates used. However, this increase should be seen in the context of equity, i.e. serving populations that were previously not benefitting from health services to the same extent as the hearing population and hence a system that could be seen as having been inequitable [22]. Further, there could be potential cost savings due to a reduction in complications associated with inadequate communication such as overdosing and non-compliance with treatment [23]. To minimise these costs, particularly the administrative costs of running such a service, a recommendation could be the incorporation of this service within the already established administrative structures in the sub districts.

There were instances where an interpreter could not be provided due to shortages. This situation is not unique to the project and neither is it unique to South Africa. The DeafSA has argued that the current interpreter ratio is not adequate to meet the needs of the Deaf population in South Africa [19]. In general, proficient SLIs are not available in sufficient numbers and in particular the SLI proficient in medical interpreting are even fewer if at all they are available [16], also seen in Ireland [24], the UK [25] and the USA [11]. However, to bridge this gap the pilot project started the SASLI training programme that led to the steady decline in unmet need as shown in this analysis. This is one way of solving this problem in the context of district health services.

Most of the requests for a SASLI were for primary healthcare visits. SASLI could be a critical part of the district health services as primary health care clinics are often the first point of contact and thus serve as the gateway to health care for many patients.

The results from this study show that most of the Deaf respondents had chosen English as the language for communication with the pilot project. This could be due to English being the “lingua franca or language of wider communication” in South Africa for historical and political reasons [26]. In addition, English is the language mostly seen by the Deaf particularly in written form through the Internet, print and electronic media. However it should be borne in mind that although most had chosen English it does not always imply competency [5,15].

The income and employment characteristics of this Deaf population illustrate a population that is materially better off than what may be expected. This could be due to the fact that the Deaf who eventually become part of any study are usually more educated and are materially better off than the average Deaf individual [3]. Further the poorer members of society are often difficult to reach in surveys[3], which might be the case in this study. In the case of this specific population, the majority of the SASLI clients were from the Southern sub district, which was previously found to have the highest socio-economic status in the Western Cape [27].

Income was collected as an indicator of socio-economic status in this study and, based on these findings, the Deaf people interviewed are unlikely to afford the services of an interpreter out of pocket as most of them had monthly incomes of less than US$415.

In a previous study, it was found that on average 62% of the adult respondents older than 20 years had not reached matric in the Cape Metropole. The highest level was in Khayelitsha with 76% [27]. While in the present study, the level was higher than either of these, at 96%, this is not surprising as has been reported elsewhere that the Deaf have higher levels of educational illiteracy [3,15,28]. However, the low literacy levels are also a concern given that the average age of these respondents was 40.5 years, a group of people in the prime of their economic productivity. Therefore, these low levels of education are likely to impact negatively on the Deaf people’s meaningful and productive participation in the economy through formal employment. It is worth noting that there were 2 people amongst the respondents who did not know their level of education. Some of this lack of knowledge could be explained by growing up and living in an overprotective environment whereby the parents of the Deaf often speak for and on behalf of their Deaf children and consequently may not share any information with the Deaf [5]. This is also a significant issue where the Deaf may not be aware of their medical history, making the consultation with a health professional less satisfactory if all the relevant medical history cannot be shared by the patient [3].

The results show that the inclusion of the SASLI within the health services in the Cape Metro district is likely to consume between 1–5% of the Provincial health budget, and 2–12% of the budget allocation for District Health services. This amount represents the cost for up-scaling this service in only one of 6 districts of the Western Cape Province. However in order to reduce the upward trajectory of costs, alternatives could include sharing of costs between the department of social services and the department of health. In addition, utilising the already set up infrastructure within the District health services may reduce capital costs. Currently, two sub districts are assigned to a single directorate for the management functions; this may potentially reduce the administrative costs. Although the costs of implementing SASLI in health care appears high, this study has not explored potential cost savings through improved communication and better compliance with medical instruction [13, 28].

The study is not without its limitations. Firstly, the sample on which the secondary data analysis was done is a non-randomised sample of Deaf respondents, which limits the external validity of the findings. This is due to using a fortuitous sample of respondents who had previously used the service or were likely to use the service and to whom project marketing was done in social gatherings. This means that the people getting the information about the SASLI project are likely to be similar and move in similar social circles much to the exclusion of those that are not associated with the organisations that convene these meetings. As such, the sampling technique used here is likely to underrepresent the poorer, less educated individuals. However, this is not unique to this study as it is often difficult to obtain a representative sample of a group of Deaf people due to issues related to researcher bias, communication challenges and mistrust between the Deaf community and the hearing individuals [3]. In addition, the income data may not be entirely accurate or reliable due to challenges in collecting income data such as monthly fluctuations, informal work and reporting biases.

Secondly, data on the number of Deaf people or Sign Language users is hard to come by. In this study, estimates from the DeafSA were used which gave a wide range of 600 000 to 1.5million SASL users. Using these estimates in calculating the costs may have under or overestimated the true population in need, which similarly influences the estimated costs of providing such a service.

Thirdly, the pilot project is housed at the University of Cape Town with full access to the amenities thereof. This could mask some of the true costs a project of this nature could have such as office space; printers etc. were it in a commercial space.

Fourthly, the average number of interpreter-assisted visits was taken to approximate the average utilisation rate of health services per person. However, this underestimates the true number of visits because it assumes the patient would not utilise the health services if an interpreter were unavailable from the pilot project. Further, not all signing Deaf patients will require an interpreter as assumed in the calculations, hence actual costs may be lower than the estimated values.

Lastly, direct extrapolation of cost results from the pilot without factoring in the economies of scale and other relevant factors that influence costs of scaling up, may influence the actual costs of running this service at scale. Further, this study did not attempt to estimate the potential costs saved by the health sector through having an interpreter present.

## Conclusions

Using SASLI in health care has the potential to improve access and utilisation of health care services amongst the Deaf. Therefore, targeted efforts at the Deaf, especially for the HIV/AIDS prevention and treatment, are likely to have a higher uptake in the presence of SASLI in health care. However, funding of this service would require government to make a significant investment, as the Deaf cannot afford out of pocket given their socio-economic circumstances.

## Supporting information

S1 Table South African Sign Language Interpreter training costs. (DOCX)

S2 Table The utilisation pattern of Sign Language Interpreters 2008–2013. (DOCX)
