# Supplementary material for: The cost and utilisation patterns of a pilot sign language interpreter service for primary health care services in South Africa
Source: PLoS One. 2017 Dec 22;12(12):e0189983. doi: 10.1371/journal.pone.0189983 (PMC5741243; doi:10.1371/journal.pone.0189983)
Supplement: S1 Table — (DOCX) [file pone.0189983.s001.docx]

S1 Table South African Sign Language Interpreter training costs

| **Course Description** | **Total Costs per participant** | **Number of participants** | **Annuitisation factor (5years, 6%)** | **Equivalent Annual cost** |
| --- | --- | --- | --- | --- |
| Level 1 (basic SLI) | $5 384.49 | 4 | 4.2124 | $5 112.99 |
| Advanced level 2 (SLI in health) | $5 384.49 | 4 | 4.2124 | $5 112.99 |
| **Total Training Costs** |  | | | **$10 225.98** |
| **Average training cost per visit** |  | | | **$29.56** |
