# Supplementary material for: The cost and utilisation patterns of a pilot sign language interpreter service for primary health care services in South Africa
Source: PLoS One. 2017 Dec 22;12(12):e0189983. doi: 10.1371/journal.pone.0189983 (PMC5741243; doi:10.1371/journal.pone.0189983)
Supplement: S2 Table — (DOCX) [file pone.0189983.s002.docx]

S2 Table The utilisation pattern of Sign Language Interpreters 2008-2013

| **Year** | **2008** | **2009** | **2010** | **2011** | **2012** | **2013** | **All** |
| --- | --- | --- | --- | --- | --- | --- | --- |
| Total requests per year | 39 | 103 | 155 | 148 | 189 | 366 | 1000 |
| Number of clients per year | 15 | 38 | 48 | 42 | 58 | 91 | 292 |
| Interpreter assisted visits | 27 | 63 | 139 | 121 | 165 | 326 | 841 |
| Unmet need (no interpreter) | 12 | 40 | 16 | 27 | 24 | 40 | 159 |
| Avge requests per person | 2.60 | 2.71 | 3.23 | 3.52 | 3.26 | 4.02 | 3.13 |
| Avge actual utilisation per person | 1.80 | 1.66 | 2.90 | 2.88 | 2.84 | 3.58 | 2.88 |
